# Aberrations in translational regulation are associated with poor prognosis in hormone receptor-positive breast cancer

**DOI:** 10.1186/bcr3343

**Published:** 2012-10-26

**Authors:** Funda Meric-Bernstam, Huiqin Chen, Argun Akcakanat, Kim-Anh Do, Ana Lluch, Bryan T Hennessy, Gabriel N Hortobagyi, Gordon B Mills, Ana Maria Gonzalez-Angulo

**Affiliations:** 1 Department of Surgical Oncology, The University of Texas MD Anderson Cancer Center, 1515 Holcombe Blvd, Houston, TX 77030, USA; 2 Department of Breast Medical Oncology, The University of Texas MD Anderson Cancer Center, 1515 Holcombe Blvd, Houston, TX 77030, USA; 3 Department of Biostatistics, The University of Texas MD Anderson Cancer Center, 1515 Holcombe Blvd, Houston, TX 77030, USA; 4 Department of Hematology and Oncology, Hospital Clinico Universitario de Valencia, Avda Blasco Ibáñez, 17, 46010 Valencia, Spain; 5 Department of Gynecologic Medical Oncology, The University of Texas MD Anderson Cancer Center, 1515 Holcombe Blvd, Houston, TX 77030, USA; 6 Department of Medical Oncology, Beaumont Hospital, Beaumont Road, Beaumont, Dublin 9, Ireland; 7 Department of Systems Biology, The University of Texas MD Anderson Cancer Center, 1515 Holcombe Blvd, Houston, TX 77030, USA

## Abstract

**Introduction:**

Translation initiation is activated in cancer through increase in eukaryotic initiation factor 4E (eIF4E), eIF4G, phosphorylated eIF4E-binding protein (p4E-BP1) and phosphorylated ribosomal protein S6 (pS6), and decreased programmed cell death protein 4 (pdcd4), a translational inhibitor. Further, translation elongation is deregulated though alterations in eukaryotic elongation factor 2 (eEF2) and eEF2 kinase (eEF2K). We sought to determine the association of these translational aberrations with clinical-pathologic factors and survival outcomes in hormone receptor-positive breast cancer.

**Methods:**

Primary tumors were collected from 190 patients with Stage I to III hormone receptor-positive breast cancer. Expression of eIF4E, eIF4G, 4E-BP1, p4E-BP1 T37/46, p4E-BP1 S65, p4E-BP1 T70, S6, pS6 S235/236, pS6 S240/244, pdcd4, eEF2 and eEF2K was assessed by reverse phase protein arrays. Univariable and multivariable analyses for recurrence-free survival (RFS) and overall survival (OS) were performed.

**Results:**

High eEF2, S6, pS6 S240/244, p4E-BP1 T70, and low pdcd4 were significantly associated with node positivity. Median follow-up for living patients was 96 months.

High p4E-BP1 T36/47, p4E-BP1 S65, p4E-BP1 T70 and 4E-BP1 were associated with worse RFS. High p4E-BP1 T70 and pS6 S235/236, and low pdcd4, were associated with worse OS. In multivariable analysis, in addition to positive nodes, p4E-BP1 S65 remained a significant predictor of RFS (HR = 1.62, 95% CI = 1.13-2.31; *P *= 0.008). In addition to age, pS6 S235/236 (HR = 1.73, 95% CI = 1.03-2.90, *P *= 0.039), eEF2K (HR = 2.19, 95% CI = 1.35-3.56, *P *= 0.002) and pdcd4 (HR = 0.42, 95% CI = 0.25-0.70, *P *= 0.001) were associated with OS.

**Conclusions:**

Increased pS6, p4E-BP1, eEF2K and decreased pdcd4 are associated with poor prognosis in hormone receptor-positive breast cancer, suggesting their role as prognostic markers and therapeutic targets.

## Introduction

**Figure 1:**
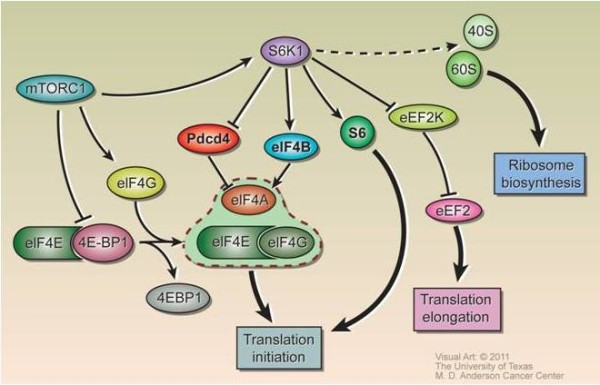
**Translational regulation**. Translation initiation and elongation is regulated through the expression and phosphorylation status of several proteins. Arrows represent activation and bars represent inhibition.

Control of mRNA translation to protein is an important point of regulation for gene expression. Translation is deregulated in cancer through a variety of mechanisms [1]. The most recognized alteration in translation is the overexpression of eukaryotic initiation factor 4E (eIF4E), the mRNA 5'cap-binding protein. Cap-dependent mRNAs initiate translation through interaction with the cap-dependent initiation complex eIF4F, comprised of eIF4E, scaffold protein eIF4G, and ATP-dependent helicase eIF4A (Figure 1) [2]. eIF4E is the rate limiting step for cap-dependent translation [3]. eIF4E overexpression leads to selective translation of a subset of mRNA such as cyclin D1, Bcl-2, Bcl-xL, and vascular endothelial growth factor, enhances nucleocytoplasmic transport for selected mRNA such as cyclin D1 and mediates Akt activation by upregulating Nijmegen breakage syndrome protein 1, an Akt pathway activator [1,3-7]. eIF4E overexpression has transforming activity in fibroblasts and mammary epithelial cells [8]. In transgenic mice, eIF4E-overexpression mice develop tumors of various histologies [9]. Thus, eIF4E also directly acts as an oncogene *in vivo*. Further, formation of the eIF4F complex determines the sensitivity to chemotherapy, as well as anticancer drugs targeting HER2 and EGFR [10].

Activated translation initiation is essential for the malignant breast cancer phenotype. eIF4E is overexpressed in breast cancer and has been suggested to be an indicator of poor prognosis [11,12]. Overproduction of eIF4G, similar to eIF4E, leads to malignant transformation *in vitro *[13]. Translation of mRNAs involved in cell growth, proliferation and bioenergetics were selectively inhibited by reduction in eIF4G1 [14]. Expression of initiation factor eIF4G is increased in locally advanced breast cancers (52%) compared to small breast cancers (11%) (52% vs. 11%, *P *= 0.0023), and the overexpression of 4E-BP1 and eIF4G have been proposed to orchestrate a hypoxia-activated switch from cap-dependent to cap-independent mRNA translation that promotes increased tumor angiogenesis and local tumor growth [15].

eIF4G1 is also overexpressed in inflammatory breast cancer, where it reprograms the translational machinery to increase translation of mRNA with internal ribosome entry sites (IRES) that promote cell survival and tumor emboli [16].

eIF4E-binding proteins (4E-BP) compete with eIF4G for a binding site in eIF4E. The binding of 4E-BP1 to eIF4E is regulated by phosphorylation; 4E-BP1 hyperphosphorylation decreases this binding, increasing eIF4E availability to engage the cap initiation complex eIF4F. 4E-BP1 is phosphorylated on multiple residues: T37, T46, S65, T70; phosphorylation at least in part is regulated through PI3K/Akt/mTOR signaling. High levels of phosphorylated eIF4E-binding protein 1 (p4E-BP1) have been associated with worse prognosis in several tumor types including breast cancer [17]. Further, additional prognostic information is gained by combining assessment of 4E-BPs with eIF4E analysis [18].

Programmed cell death protein 4 (pdcd4) is a tumor suppressor protein that inhibits breast cancer cell invasion. Pdcd4 inhibits protein translation by binding to the translation initiation factor eIF4A. Pdcd4 is targeted for degradation during tumor promotion [19]. Pdcd4 undergoes regulated degradation by β-Trcp after phosphorylation at S67 by S6K1 [20].

Ribosomal protein S6 (S6) is a component of the 40S ribosomal subunit that mediates translation initiation. In response to mitogenic stimuli, S6 undergoes phosphorylation by S6K1 and p90 ribosomal S6 kinases on four serine residues (S235, S236, S240, and S244); these modifications potentiate S6 cap-binding activity. S6 phosphorylation correlates with increased translation of mRNA with 5' terminal oligopyrimidine tracts in some studies conditions, but not in others. S6 is also proposed to subsequently undergo casein kinase1-dependent phosphorylation of S247; phosphorylation of S6 promotes its association with the mRNA cap-binding complex *in vitro *[21]. Thus S6's role in translation may be cell, tissue or context-specific.

Eukaryotic elongation factor-2 kinase (eEF2K) phosphorylates and inactivates eukaryotic elongation factor 2 (eEF2), an elongation factor that controls the rate of peptide chain elongation. The activity of eEF2 is increased in several tumor types including breast cancer [22,23]. eEF2K also plays a regulatory role in autophagy, and inhibitors of eEF2K promote cell death [24,25]. eEF2K/eEF2 signaling may promote cell survival by decreasing energy utilization on protein synthesis in conditions of stress such as nutrient deprivation or hypoxia and regulating autophagy [25].

Thus, taken together, a significant amount of data has accumulated suggesting an important role for translational dysregulation in breast cancer. It remains unclear, however, which of these alterations are the most significant determinants of cancer progression and poor oncologic outcomes. We sought to determine the association of translational regulators with clinical-pathologic factors and survival outcomes in hormone receptor-positive breast cancer.

## Materials and methods

### Patient samples

Primary tumors were collected from 190 patients with Stage I to III hormone receptor-positive breast cancer treated at Hospital Clinico Universitario de Valencia, Spain. Tumors were collected from surgical samples, and tumor content verified by histopathology. Patient cohort was selected based on hormone receptor-positive status, availability of adequate frozen tissue, and subsequent treatment limited to endocrine therapy. All tissues were collected after informed consent for future research. The study was approved by the MD Anderson Cancer Center Institutional Review Board as well as by Hospital Clinico Universitario de Valencia. Tumors were characterized for estrogen receptor (ER) and progesterone receptor (PR) status by immunohistochemistry. ER/PR positivity was designated when nuclear staining occurred in ≥10% of tumor cells. Hormone receptor positivity was designated when either ER or PR was positive. All patients were treated with adjuvant endocrine therapy (tamoxifen); none received chemotherapy. HER2 testing was not routinely performed; none of the patients received HER2-targeted therapy. None of the patients received neoadjuvant therapy.

### Reverse phase protein arrays

Reverse phase protein arrays (RPPA) was performed in the MD Anderson Cancer Center Functional Proteomics RPPA Facility as described previously [26,27]. Briefly, tumor samples homogenized in cold lysis buffer (50 mmol/L HEPES, pH 7.4; 150 mmol/L NaCl; 1% Triton X-100; 1 mmol/L EGTA; 100 mmol/L NaF; 10 mmol/L sodium pyrophosphate; 1 mmol/L Na_3_VO_4_; 10% glycerol, 1x complete protease inhibitor cocktail (Roche Applied Science, Indianapolis, IN)). After centrifugation, supernatant was transferred to a fresh tube and protein concentration was corrected to 1 μg/μL.

The supernatants were subsequently manually diluted in five-fold serial dilutions with lysis buffer. An Aushon Biosystems (Burlington, MA, USA) 2470 Arrayer created 1,056 sample arrays on nitrocellulose-coated FAST slides (Schleicher & Schuell BioScience, Inc., Keene, NH, USA) from the serial dilutions. Slides were then probed with primary antibodies including eIF4E, eIF4G, 4E-BP1, p4E-BP1 T37/46, p4E-BP1 S65, p4E-BP1 T70, S6, pS6 S235/236, pS6 S240/244, pdcd4, eEF2 and eEF2K (Table 1 in Additional file 1). The signal was amplified using a DakoCytomation-catalyzed system (Dako North America, Inc., Carpinteria, CA, USA). Secondary antibodies were used as a starting point for amplification. The slides were scanned, analyzed, and quantitated using MicroVigene software (VigeneTech Inc., Carlisle, MA, USA) to generate serial dilution-signal intensity curves for each sample, and processed by the R package SuperCurve (version 1.01) [28]. A fitted curve (called 'supercurve') was plotted with the signal intensities on the Y-axis and the relative log2 concentration of each protein on the X-axis using the nonparametric, monotone increasing B-spline model [28]. The protein concentrations were derived from the supercurve for each sample lysate on the slide by curve fitting and then normalized by median polish. Each protein measurement was subsequently corrected for loading as previously described [26].

**Table 1:** Patient and tumor characteristics.

	Overall	
	**N = 190**	**%**

**Age at diagnosis**		

>50	170	89.5%
≤50	20	10.5%
Median	68	
Range	30-89	

**Nodal status**		

Positive	68	35.8%
Negative	122	64.2%

**Stage**		

I	47	24.7%
II	119	62.6%
III	24	12.6%

**Histology**		

Ductal	171	90.0%
Other	19	10.0%

**T stage**		

T1	64	33.7%
T2-T4	122	64.2%

**Grade^a^**		

I	49	43.0%
II	58	50.9%
III	7	6.1%

**ER**		

Positive	186	97.9%
Negative**^b^**	4	2.1%

**PR**		

Positive	140	73.7%
Negative	50	34.2%

**^a^**Distribution of grade among patients where grade was available; **^b^**four patients were estrogen receptor (ER)-negative but progesterone receptor (PR)-positive, thus hormone receptor-positive.

### Statistical analysis

RPPA data from 190 hormone receptor-positive and Stage I to III patients was median-polish normalized. The samples were tabulated and described according to their clinical characteristics. Two sample *t *tests were applied to examine the differential expression/phosphorylation of translational factors between stage I and II/III tumors; their means and standard deviations were also provided. The same method was used to detect the factors differentially expressed between node-positive and -negative patients. To adjust for multiple comparisons, false discovery rate (FDR) was calculated by using the R package 'fdrtool'. Box plots were used to display the significant proteins. The FDR threshold of 0.05 was used for declaration of significance.

For each of the proteins of interest, a univariable CoxPH model was established for both recurrence-free survival (RFS) and overall survival (OS). For multivariable analysis, a boosting approach (R package CoxBoost) was employed to develop a Cox proportional hazard model, and to select predictors. Beside age, nodal status, and T stage, all other factors were treated as optional covariates. As the main model complexity parameter, the number of boosting steps, stepno, was selected with cross-validation by using cv.CoxBoost. The penalty parameter was chosen by using optimCoxBoostPenalty. The predictors with estimated nonzero coefficients were considered to be incorporated in the final multivariable model. Age, nodal status and T stage were also retained as mandatory covariates in the final model because of their clinical significance. The final model selection was undertaken based on a backwards selection procedure, during which all factors of interest identified by CoxBoost were incorporated in a full model and then variables were retained according to their *P *values (*P *<0.05).

In order to divide the patients into two groups (high and low) based on expression levels of factors in the final multivariate model, a regression tree method of R package 'rpart' was applied to find the best cutoff points for p4E-BP1 S65, pS6 S235/236, eEF2K and pdcd4. Five-year survivals for RFS or OS were estimated between each protein's high and low expression groups. Logrank tests were used to evaluate statistical significance. Kaplan-Meier curves by expression level group were presented as well. *P *values less than 0.05 were considered statistically significant and all tests were two-sided. All statistical analysis has been done with R statistical software version 2.11.0.

## Results

### Association between translational regulators and clinical-pathologic characteristics

The patient characteristics are shown in Table 1. The majority (89.7%) of the patients were older than 50 years of age, and the median age was 68 (range 30 to 89). Most patients (64.2%) had node-negative breast cancer. Most patients had T2 or greater disease (64.2%).

Next, we determined whether expression/phosphorylation of specific translational factors correlated with clinical-pathologic characteristics. Table S2 in Additional file 1 demonstrates the association between translational factors and T stage. Only eEF2 expression showed a significant association with tumor size (mean expression in T1: 1.89 arbitrary units vs. mean of T2 to 4 tumors: 2.11, *P *= 0.016).

**Figure 2:**
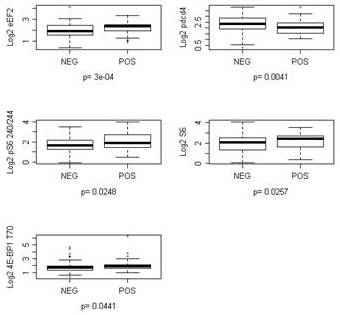
**Proteins differentially expressed (FDR <0.2) by nodal status in hormone receptor-positive patients**.

Next, we determined association between translational regulators and axillary nodal status. Higher expression of eEF2, S6, pS6 S240/244 and p4E-BP1 T70 was significantly associated with node positivity (FDR <0.2; Figure 2). On the contrary, lower expression of pdcd4 was associated with node positivity. Table 3 in Additional file 1 demonstrates the association between translational regulators and nodal status.

### Translational regulators and recurrence-free and overall survival

At a median follow-up of 87 months (range 1 to 197 months) there were 47 recurrences and 65 deaths. The median follow-up for living patients was 96 months. In order to identify predictive factors, a Cox proportional hazard model including all of the 14 factors as optional predictors had been established at first. For each of the proteins of interest, a univariable CoxPH model results for both RFS and OS are displayed in Table 2. Interestingly, high p4E-BP1 T36/47, p4E-BP1 S65, p4E-BP1 T70 as well as total 4E-BP1 were associated with worse RFS. This may appear paradoxical as p4E-BP1 would be expected to increase translation, and increased 4E-BP1 would be expected to decrease it. However, these markers are not independent from each other for at least two reasons: increased total 4E-BP1 may be associated with higher levels of p4E-BP1, and eIF4E levels and availability may regulate expression of 4E-BP1 [8,29].

**Table 2:** Univariate analysis of RFS and OS for hormone receptor-positive breast cancer patients.

Recurrence-free survival				
**Variable**	**HR**	**95% CI**	***P *value**	**FDR**

Translational regulator				

p4E-BP1 S65	1.51	1.08-2.11	0.02	0.09
p4E-BP1 T36/47	1.49	1.05-2.12	0.03	0.09
4E-BP1	1.66	1.04-2.65	0.04	0.09
p4E-BP1 T70	1.38	1.00-1.90	0.05	0.10
pS6 S235/236	1.37	0.81-2.31	0.23	0.27
pS6 S240/244	1.23	0.83-1.83	0.30	0.29
eIF4G	1.33	0.77-2.30	0.30	0.30
Pdcd4	0.81	0.52-1.26	0.34	0.31
eIF4E	1.12	0.75-1.67	0.58	0.43
S6	0.96	0.67-1.37	0.82	0.52
eEF2K	1.04	0.70-1.55	0.83	0.52
eEF2	0.98	0.59-1.61	0.93	0.55
				

Age at diagnosis	1.01	0.98-1.04	0.64	
				

Nodal Status				

Negative	1			
Positive	2.789	1.55-5.03	<0.01	
				

T Stage				

T1	1			
T2-4	1.339	0.71-2.52	0.37	
				

**Overall survival**				

**Variable**	**HR**	**95% CI**		**FDR**

Translational regulator				

Pdcd4	0.62	0.42-0.91	0.0155	0.06
p4E-BP1 T70	1.30	0.98-1.71	0.0662	0.12
pS6 S235/236	1.40	0.90-2.18	0.1369	0.14
eEF2	1.27	0.82-1.95	0.2852	0.15
4EBP1	1.22	0.78-1.91	0.3801	0.16
eEF2K	1.15	0.82-1.62	0.4093	0.16
p4E-BP1 S65	1.15	0.82-1.60	0.4144	0.16
S6	1.14	0.83-1.55	0.4192	0.16
pS6 S240/244	1.14	0.81-1.60	0.4484	0.16
p4E-BP1 T36/47	1.13	0.81-1.56	0.4670	0.16
eIF4E	0.94	0.68-1.31	0.7162	0.22
eIF4G	0.98	0.61-1.55	0.9142	0.27
				

Age at diagnosis	1.05	1.03-1.08	<0.01	
				

Nodal status				

Negative	1			
Positive	2.05	1.25-3.35	<0.01	
				

T Stage				

T1	1			
T2-T4	1.36	0.79-2.34	0.26	

CI, confidence interval; FDR, false discovery rate; HR, hazard ratio.

A boosting approach (R package CoxBoost) is applied to determine the corresponding significance. Next, a full multivariate model has been developed that incorporates all the factors, which have survived from the boosting approach and clinic variables based on their statistical or clinical significance. The final model selection is undertaken through a backwards selection procedure, during which the factors of interests are retained if their *P *values are less than 0.05 (*P *<0.05).

**Figure 3:**
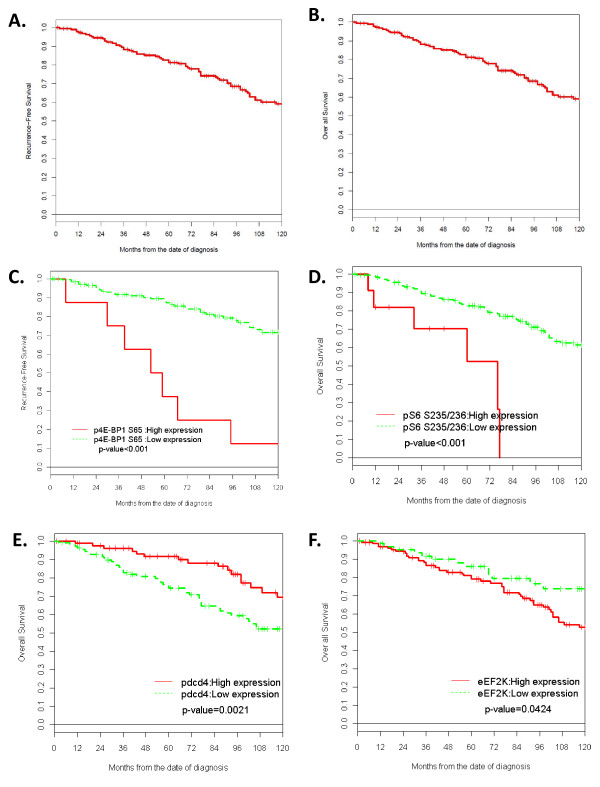
**Kaplan-Meier analysis of recurrence-free and overall survival**. **(A) **Recurrence-free survival of the entire cohort. **(B) **Overall survival of the entire cohort. **(C) **Recurrence-free survival in patients by p4E-BP1 S65 expression. High: p4E-BP1 S65 >3.162, Low: p4E-BP1 S65 ≤3.162 (arbitrary units). **(D) **Overall survival of patients by pS6 S235/236 expression levels. High: pS6 S235/236 >2.75, Low: pS6 S235/236 ≤2.75. **(E) **Overall survival of patients by pdcd4 expression levels. High: pdcd4 >2.357, Low: pdcd4 ≤2.357. **(F) **Overall of patients by eEF2K expression levels. High: eEF2K >2.248, Low: eEF2K ≤2.248.

When age, nodal status and T stage were added to the model, in addition to positive nodes, p4E-BP1 S65 remained a significant predictor of RFS (hazard ratio (HR) = 1.62, 95% confidence interval (CI) = 1.13-2.31, *P *= 0.008). The final multivariable models of RFS and OS are presented in Table 3. The five-year RFS was significantly different between patients with high and low expression of p4E-BP1 S65 (37.5% vs. 88.7%, *P *≤0.001, Figure 3, Table 4). There were no differences between the expression of the translational regulators tested between patients who had recurrences early (within two years) vs. late (after five years).

**Table 3:** Multivariable analysis of RFS and OS for hormone receptor-positive breast cancer patients.

Recurrence-free survival			
**Variable**	**HR**	**95% CI**	***P *value**

Age at diagnosis	1.00	0.97-1.03	0.951

Nodal status			

Negative	1		
Positive	3.08	1.66-5.73	<0.001

T stage			

T1	1		
T2-4	1.31	0.66-2.58	0.439

p4E-BP1 S65	1.62	1.13-2.31	0.008

**Overall survival**			

**Variable**	**HR**	**95% CI**	***P *value**

Age at diagnosis	1.04	1.01-1.07	0.003

Nodal status			

Negative			
Positive	1.29	0.75-2.23	0.355

T stage			

T1			
T2-4	1.02	0.58-1.81	0.945

pS6 S235/236	1.73	1.03-2.90	0.039

eEF2K	2.19	1.35-3.56	0.002

Pdcd4	0.42	0.25-0.70	0.001

CI, confidence interval; HR, hazard ratio.

**Table 4:** Five-year survival estimates by expression levels of translational regulators.

		N	Events	Five-year estimate	95% CI	*P *value
**RFS**						

p4E-BP1 S65	High	8	7	37.5%	(15.3%, 91.7%)	
	Low	180	40	88.7%	(83.9%, 93.8%)	<0.0001

**OS**						

pS6 S235/236	High	11	6	52.6%	(26.1%, 100%)	
	Low	177	59	87.9%	(82.7%, 88.8%)	0.0001

eEF2K	High	126	51	79.0%	(71.8%, 86.9%)	
	Low	62	14	85.9%	(77.3%, 95.5%)	0.0424

Pdcd4	High	78	16	91.5%	(85.2%, 98.3%)	
	Low	110	49	74.2%	(66.2%, 83.4%)	0.0021

CI, confidence interval; OS, overall survival; RFS, recurrence-free survival.

In addition to age, three translational regulators were associated with OS on the multivariable model; these were pS6 S235/236 (HR = 1.73, *P *= 0.039), eEF2K (HR = 2.19, *P *= 0.002) and pdcd4 (HR = 0.42, *P *= 0.001) (Table 3). Classification by expression of pS6 S235/236, eEF2K or pdcd4 resulted in patient groups with significantly different five-year OS: pS6 S235/236 high 52.6% vs. low 87.9%, *P *<0.001; eEF2K high 79.0% vs. low 85.9%, *P *= 0.0424; pdcd4 high 91.5% vs. low 74.2%: *P *= 0.0021 (Figure 3). The five-year survival estimates and logrank test results are listed in Table 4.

## Discussion

A significant amount of data has accumulated suggesting an important role for translational dysregulation in many cancer lineages, including breast cancer. It remained unclear, however, which of these alterations are the most significant determinants of cancer progression and poor patient outcomes. We sought to determine the association of translational regulators with clinical-pathologic factors and survival outcomes in hormone receptor-positive breast cancer. We found that high eEF2, S6, pS6 S240/244, p4E-BP1 T70, and low pdcd4 were significantly associated with node positivity. High p4E-BP1 T36/47, p4E-BP1 S65, p4E-BP1 T70 as well as total 4E-BP1 were associated with worse RFS. High p4E-BP1 T70 and pS6 S235/236, and low pdcd4, were associated with worse OS. In the multivariable analysis, in addition to positive nodes, high p4E-BP1 S65 remained a significant predictor of lower RFS. High pS6 S235/236, eEF2K and low pdcd4 were associated with lower OS. These results confirm that translational dysregulation plays an important role in breast cancer progression and relapse suggesting a role for these as prognostic markers as well as therapeutic targets.

Our results support the role of PI3K/mTOR pathway inhibitors for breast cancer treatment in HR-positive breast cancer. The PI3K/mTOR signaling pathway controls phosphorylation of 4E-BP1 and S6K, and S6K1 also phosphorylates S6, and has been proposed to phosphorylate EF2K and pdcd4, controlling the activity of S6 and EF2K, and protein stability of pdcd4 [20,30-32]. S6K1 also regulates ERα activation through S167 phosphorylation [33], adding to the growing evidence that there is cross-talk between ER and PI3K/mTOR signaling. Our results are consistent with that of Miller *et al*., who have shown that in 64 hormone receptor-positive breast cancer patients, a baseline signature of PI3K pathway activation is predictive of poor outcome after adjuvant endocrine therapy [34]. Recently, ER-positive tumors with long-term estrogen deprivation have been shown to exhibit increased PI3K/mTOR signaling [34]. Preclinical studies have demonstrated that the antitumor efficacy of tamoxifen, fulvestrant as well as estrogen deprivation can be enhanced with inhibition of PI3K/mTOR signaling [1]. A phase II trial of tamoxifen with and without the rapamycin analog everolimus has shown significant improvement in progression-free survival (PFS) in hormone receptor-positive patients [35]. A phase II neoadjuvant trial of letrozole with or without everolimus showed greater inhibition of cell proliferation (Ki-67) with the everolimus combination at two weeks, as well as a greater clinical response rate [35]. Recently, the exciting data from the phase III BOLERO-2 trial was released, demonstrating a significantly greater PFS with the combination of exemestane and everolimus compared with exemestane alone [36]. Studies are ongoing with endocrine therapy in combination with new inhibitors of PI3K, Akt and PI3K/mTOR dual inhibitors.

There are several approaches to inhibit translation in preclinical development. As a proof of concept, in previous work we have shown that eIF4E siRNA knockdown inhibits cancer cell growth in a variety of breast cancer cell subtypes [29]. Graff *et al*. have shown that eIF4E downregulation with second-generation antisense oligonucleotides reduces *in vivo *tumor growth in a PC-3 prostate cancer model and MDA-MB-231 breast cancer model [37]. A phase I trial of antisense oligonucleotides targeting eIF4E has recently been completed [38]. eIF4E-binding motif peptides can also interfere with eIF4E-eIF4G binding, translation initiation, cell cycle, and survival, providing proof of concept that eIF4E-binding small-molecule inhibitors may have utility in cancer therapy [39,40]. A 4E-BP1-based peptide fused to a GnRH agonist was shown to be taken up by GnRHRI-expressing ovarian cancer cells and inhibit growth *in vitro *and *in vivo *[41].

Moerke *et al*. identified inhibitors of the eIF4E/eIF4G interaction in a high-throughput screen [42]. The most potent compound exhibited *in vitro *activity against multiple cancer cell lines and appeared to have preferential effect on transformed cells [42]. Cencic *et al*. reported that eIF4E:eIF4G interaction inhibitors can reverse tumor chemoresistance in lymphoma models [43]. Another potential approach to inhibit translation is by interfering with eIF4E binding to the 7-methyl guanosine cap or by interfering with eIF4E binding to the multidomain adaptor protein eIF4G, thus interfering with assembly of the translation initiation complex eIF4F. Kentsis *et al*. reported that the antiviral guanosine analog ribovirin binds to eIF4E at the site used by the 7-methyl guanosine cap, competing with eIF4E binding and disrupting the transport and translation of mRNAs regulated by eIF4E [44]. Thus, translation initiation is actively being pursued as a therapeutic target. As activation of translation initiation is a common integral pathway for the malignant phenotype, these approaches may hold promise for a variety of tumor types.

Pdcd4 has been reported to inhibit protein translation by binding to the translation initiation factor eIF4A [45]. As pdcd4 undergoes regulated degradation by β-Trcp after phosphorylation at S67 by S6K1 [20], PI3K/mTOR pathway inhibitors may increase pdcd4 expression at least in some cancer cell lines. However, targeting eIF4E directly may provide an alternate strategy for pdcd4-low tumors. Pateamine A, a marine natural product with potent antiproliferative and immunosuppressive activities, was also found to inhibit protein translation, inhibiting the eukaryotic eIF4A family of RNA helicases [46]. Des-methyl, des-amino pateamine A (DMDA-PatA), a structurally simplified analogue of pateamine A, was recently shown to have potent antiproliferative activity against a wide variety of human cancer cell lines [47]. However, it is of note that pdcd4 has other tumor-suppressive functions reported such as inhibiting AP-1 transactivation [48], and thus pdcd4's antitumor effect may not be limited to its effects on translation.

eEF2K is phosphorylated and inhibited by SAPK (JNK) (on S359), RSK and S6K1 (on S366), and phosphorylated and activated by AMPK (on S398) [49]. Thus eEF2K integrates a variety of diverse signaling pathways, and potentially may be targeted through different strategies. Of note, insulin signaling and serum stimulation causes downregulation of eEF2 phosphorylation and eEF2K activity, and this is blocked by rapamycin [50]. High doses of temsirolimus are accompanied by a rapid increase in phosphorylation of eEF2, but this may involve a S6K1-independent mechanism as it appears to not correlate with S6K1 activity or eEF2K S366 phosphorylation [51]. Further study is needed to determine the effect of clinically relevant doses of PI3K/mTOR pathway inhibitors *in vivo*, and their effect on eEF2K low- and high-expressing hormone receptor-positive breast cancers. NH125, a derivative of 2-methylimidazolium iodide has been identified as a potent and relatively specific inhibitor of EF2K; it was shown to have *in vitro *anticancer activity against cell lines of a variety of tumor lineages including breast cancer [24]. Thus, EF2K/EF2 activity may hold promise as a novel therapeutic target in hormone receptor-positive breast cancer.

Our study has some limitations. All patients in this study received endocrine therapy. Thus, it is not possible to determine whether p4E-BP1, pS6, eEF2K and pdcd4 are associated with prognosis or whether they are associated with endocrine therapy resistance. Patients received adjuvant tamoxifen; however, the exact duration of adjuvant treatment with tamoxifen, and compliance with the medication, is unknown. Further work is needed to determine whether these markers are also prognostic in patients who received aromatase inhibitors. We do not have detailed information on locoregional management including radiation therapy, and sites of relapse including locoregional recurrence, thus we are unable to dissect the role of translational regulators on locoregional control. We did not have information on comorbidities and cause of death, thus we are able to determine recurrence-free survival and overall survival but not disease-specific survival. Information on treatment after relapse was also not available. Further work is also ongoing to determine the intratumoral heterogeneity and reproducibility of these markers, and to transition these markers to alternate platforms such immunohistochemistry and multiplex proteomics assay such as Luminex or ELISA that may be used clinically to identify patients who would have a poor prognosis if treated with endocrine therapy alone. Another limitation of our study is that we do not have transcriptional profiling data from the same patients; this is especially relevant as eIF4E mRNA was shown to be prognostic in luminal B cases, and not in other subtypes [52]. It will also be important to determine how our biomarkers correlate with other RNA profile-based molecular tools to predict prognosis including those that are currently in clinical use such as Oncotype DX (Genomic Health, Inc., Redwood City, CA, USA) [53] and MammaPrint (Agendia Inc., Irvine, CA, USA) [54,55], as well as those in clinical development such as the PAM50 intrinsic subtype [56]. To obtain a cohort of patients treated with endocrine therapy only and with adequate follow-up, we elected a cohort of patients who was treated prior to these tools became widely utilized. Oncotype DX, MammaPrint and PAM50 were developed to identify patients that will have good prognosis with endocrine therapy alone, or alternately those patients that are at higher risk of relapse and thus may be offered chemotherapy followed by endocrine therapy. However, assessment of p4E-BP1, pS6, eEF2K and pdcd4 may have additional utility as these biomarkers may not only have prognostic implications, by providing biological insights. Further study is needed to determine whether these biomarkers may be used to guide specific targeted therapy selection.

## Conclusions

In summary, increased pS6, p4E-BP1, eEF2K and decreased pdcd4 are associated with poor prognosis in hormone receptor-positive breast cancer. Further study is needed to determine the clinical utility of these as prognostic or predictive markers. Our results provide further support for a role for PI3K/mTOR pathway inhibitors in the treatment of hormone receptor-positive breast cancer. The best approach to personalize treatment in hormone receptor-positive breast cancer patients with translational aberrations warrants further study.

## Abbreviations

CI: confidence interval; eEF2: eukaryotic elongation factor 2; eEF2K: eukaryotic elongation factor 2 kinase; eIF4E: eukaryotic initiation factor 4E; eIF4G: eukaryotic initiation factor 4G; ER: estrogen receptor; FDR: false discovery rate; HR: hazard ratio; OS: overall survival; p4E-BP1: phosphorylated eIF4E-binding protein; PR: progesterone receptor; PFS: progression-free survival; S6: ribosomal protein S6; pS6: phosphorylated ribosomal protein S6; pdcd4: programmed cell death protein 4; RFS: recurrence-free survival; RPPA: reverse phase protein array.

## Competing interests

This work was presented as an oral presentation at the American Association of Cancer Research 2012 Annual Meeting. The manuscript has never been published and is not under consideration for publication elsewhere. The authors have no financial interest to declare.

## Authors' contributions

FMB conceived of the study, performed data analysis and drafted the manuscript. HC performed data analysis and assisted with manuscript preparation. AA screened and optimized antibodies for RPPA and performed the RPPA. KAD supervised analysis and assisted with manuscript draft. AL identified patient samples and extracted clinical data. BTH prepared tumor lysates and supervised RPPA and clinical data coordination. GNH supervised analysis and assisted in data interpretation. GBM supervised coordination of samples, RPPA and assisted in data interpretation. AMGA assisted with specimen and data coordination, and data interpretation. All authors read and approved the manuscript.

## Supplementary Material

Additional file 1 **Supplementary tables**. **Table S1**: Antibodies used in the study. **Table S2**: Translational regulators by T stage in hormone receptor-positive breast cancer patients. **Table S3**: Translational regulators by nodal status in hormone receptor-positive breast cancer patients. Click here for file
