# Supplementary material for: Aberrations in translational regulation are associated with poor prognosis in hormone receptor-positive breast cancer
Source: Breast Cancer Res. 2012 Oct 26;14(5):R138. doi: 10.1186/bcr3343 (PMC4053117; doi:10.1186/bcr3343)
Supplement: Additional file 1 — Supplementary tables. Table S1: Antibodies used in the study. Table S2: Translational regulators by T stage in hormone receptor-positive breast cancer patients. Table S3: Translational regulators by nodal status in hormone receptor-positive breast cancer patients. [file bcr3343-S1.DOC]

**Supplementary Tables**

**Supplementary Table S1: Antibodies used in the study**

| **Antibody** | **Vendor^a^** | **Catalog number** | **RPPA dilution** |
| --- | --- | --- | --- |
| eIF4E | CST | 9742 | 200 |
| eIF4G | CST | 2498 | 100 |
| 4E-BP1 | CST | 9452 | 100 |
| p4E-BP1 T37/46 | CST | 9459 | 100 |
| p4E-BP1 S65 | CST | 9456 | 100 |
| p4E-BP1 T70 | CST | 9455 | 100 |
| S6 | CST | 2217 | 250 |
| pS6 S235/236 | CST | 2211 | 4000 |
| pS6 S240/244 | CST | 2215 | 1000 |
| Pdcd4 | Rockland | 600-401-965 | 400 |
| eEF2 | CST | 2332 | 200 |
| eEF2K | CST | 3692 | 100 |

^a^CST = Cell Signaling Technology, Inc. (Beverly, MA), Rockland = Rockland Immunochemicals (Gilbertsville, PA).

**Supplementary Table S2: Translational regulators by T stage in HR-positive breast cancer patients**

|  |  |  |  | **T1 (N=64)** | | **T2-4 (N=122)** | |
| --- | --- | --- | --- | --- | --- | --- | --- |
| **Variable** | **T stat.** | **P value** | **FDR** | **Mean** | **SD** | **Mean** | **SD** |
| eEF2 | -2.4458 | 0.0157 | 0.19 | 1.89 | 0.57 | 2.11 | 0.61 |
| S6 | -1.6049 | 0.1110 | 0.47 | 1.90 | 0.80 | 2.09 | 0.79 |
| p4E-BP1 T70 | -0.1780 | 0.8591 | 0.84 | 1.79 | 0.87 | 1.81 | 0.58 |
| 4E-BP1 | -0.1220 | 0.9031 | 0.85 | 1.65 | 0.57 | 1.66 | 0.45 |
| eIF4G | 0.1718 | 0.8639 | 0.84 | 1.83 | 0.59 | 1.81 | 0.52 |
| eIF4E | 0.2446 | 0.8072 | 0.84 | 1.70 | 0.77 | 1.65 | 0.70 |
| eEF2K | 0.6460 | 0.5194 | 0.77 | 2.37 | 0.70 | 2.30 | 0.73 |
| Pdcd4 | 0.7286 | 0.4676 | 0.75 | 2.24 | 0.70 | 2.16 | 0.66 |
| p4E-BP1 S36/47 | 0.8535 | 0.3953 | 0.71 | 1.97 | 0.82 | 1.87 | 0.66 |
| pS6 S240/244 | 0.9079 | 0.3657 | 0.70 | 1.89 | 0.76 | 1.78 | 0.74 |
| pS6 S235/236 | 1.3786 | 0.1702 | 0.52 | 1.87 | 0.53 | 1.76 | 0.60 |
| p4E-BP1 S65 | 1.5739 | 0.1185 | 0.48 | 1.98 | 0.77 | 1.80 | 0.63 |

**Supplementary Table S3: Translational regulators by nodal status in HR-positive breast cancer patients**

|  |  |  |  | **Node Negative**  **(N=122)** | | **Node Positive**  **(N=68)** | |
| --- | --- | --- | --- | --- | --- | --- | --- |
| **Variable** | **T stat.** | **P value** | **FDR** | **Mean** | **SD** | **Mean** | **SD** |
| eEF2 | -3.6630 | 0.0003 | 0.00 | 1.92 | 0.62 | 2.23 | 0.52 |
| Pdcd4 | 2.9110 | 0.0041 | 0.02 | 2.29 | 0.71 | 2.02 | 0.55 |
| pS6 S240/244 | -2.2720 | 0.0248 | 0.05 | 1.74 | 0.71 | 2.01 | 0.81 |
| S6 | -2.2544 | 0.0257 | 0.05 | 1.95 | 0.81 | 2.21 | 0.77 |
| p4E-BP1 T70 | -2.0334 | 0.0441 | 0.07 | 1.74 | 0.67 | 1.96 | 0.76 |
| pS6 S235/236 | -1.6923 | 0.0927 | 0.11 | 1.76 | 0.62 | 1.92 | 0.57 |
| eEF2K | 1.5371 | 0.1262 | 0.14 | 2.37 | 0.76 | 2.21 | 0.64 |
| 4E-BP1 | -1.1395 | 0.2567 | 0.25 | 1.62 | 0.47 | 1.71 | 0.54 |
| p4E-BP1 S65 | 0.6968 | 0.4873 | 0.39 | 1.91 | 0.63 | 1.83 | 0.78 |
| eIF4G | -0.4366 | 0.6631 | 0.46 | 1.80 | 0.55 | 1.84 | 0.55 |
| eIF4E | 0.3376 | 0.7362 | 0.49 | 1.65 | 0.73 | 1.62 | 0.73 |
| p4E-BP1 T36/47 | 0.1466 | 0.8837 | 0.53 | 1.93 | 0.65 | 1.91 | 0.84 |
